# Choline and DHA in Maternal and Infant Nutrition: Synergistic Implications in Brain and Eye Health

**DOI:** 10.3390/nu11051125

**Published:** 2019-05-21

**Authors:** Jonathan G. Mun, LeeCole L. Legette, Chioma J. Ikonte, Susan H. Mitmesser

**Affiliations:** Science & Technology, Pharmavite LLC, West Hills, CA 91304, USA; llegette@pharmavite.net (L.L.L.); cikonte@pharmavite.net (C.J.I.); smitmesser@pharmavite.net (S.H.M.)

**Keywords:** choline, pregnancy, infant nutrition, brain health, docosahexaenoic acid, DHA, eye function

## Abstract

The aim of this review is to highlight current insights into the roles of choline and docosahexaenoic acid (DHA) in maternal and infant nutrition, with special emphasis on dietary recommendations, gaps in dietary intake, and synergistic implications of both nutrients in infant brain and eye development. Adequate choline and DHA intakes are not being met by the vast majority of US adults, and even more so by women of child-bearing age. Choline and DHA play a significant role in infant brain and eye development, with inadequate intakes leading to visual and neurocognitive deficits. Emerging findings illustrate synergistic interactions between choline and DHA, indicating that insufficient intakes of one or both could have lifelong deleterious impacts on both maternal and infant health.

## 1. Introduction

Pregnancy is an important time for both mother and baby and a long-standing strategy in supporting healthy pregnancies involves adequate prenatal care. Health care during pregnancy is one of the most frequently used wellness services in the US, with more than 18 million prenatal visits in 2015 [1]. Increasing the percentage of pregnant women who receive early and adequate prenatal care is a key objective for the US Healthy People 2020 public health initiative [2]. As part of adequate prenatal health consciousness and self-care, optimal nutrition plays a key role in maternal and infant health, prior to, during, and after pregnancy. Leading health organizations including the American Congress of Obstetricians and Gynecologists (ACOG), American Academy of Pediatrics (AAP), Europe Food Safety Authority (EFSA), and the World Health Organization (WHO) have highlighted several key nutrients as vital during pregnancy, including choline and DHA. The 2018 AAP policy statement emphasized the role of nutrition in the first 1000 days of life. The AAP recognized choline and long-chain polyunsaturated fatty acids (LC-PUFAs) as key nutrients in supporting early neurodevelopment and lifelong mental health [3]. Both docosahexaenoic acid (DHA) and arachidonic acid (ARA) are common LC-PUFAs that accumulate in tissues and are known for their importance in brain function and development [4,5]. Docosahexaenoic acid and ARA are synthesized from precursor essential fatty acids, omega-3 α-linolenic acid (ALA) and omega-6 linoleic acid (LA), respectively [6]. Endogenous synthesis of choline and DHA is insufficient to meet nutrient demands, reinforcing the need for adequate daily intake. While general dietary intake of omega-6s meets daily recommendations, omega-3 intake is low in the American diet [7]. National survey data in the US population indicates inadequate dietary intakes of choline and DHA. Over 90% of the US population does not meet adequate intake for choline (550 mg/day for men, 425 mg/day for women) [8]. Among US adults, pregnant and lactating women are at increased risk for choline insufficiency as their requirements increase to 450 and 550 mg, respectively, during these life stages [9]. Dietary intakes of DHA are also low in American diet, with general recommendations of 7–8 oz of fish and seafood weekly to equate to 250 mg EPA + DHA omega-3 fatty acids daily. However, the majority of US adults fail to consume adequate amounts of seafood, with averages of 0.43 oz seafood (63 mg DHA) daily [10]. Docosahexaenoic acid recommendations for pregnant and lactating women are slightly higher, with the 2015 US Department of Agriculture Dietary Guidelines advising weekly consumption of 8–12 oz of low-mercury content seafood, noting that DHA is associated with improved infant health outcomes [11]. However, nearly all US women of child-bearing age and pregnant women (95%) have low seafood consumption and do not meet recommended daily intakes of 250 mg total of eicosapentaenoic acid (EPA) and DHA [12]. While supplement use has increased DHA and EPA intakes over time, current intakes are still low, suggesting a need for more effective nutrition education programs to address nutrient shortfalls [12], especially during pregnancy and lactation. Choline and DHA, as a LC-PUFA, are essential nutrients for normal brain development as they are integral structural components of neurological systems, and deficits during early life can have long-term impacts on brain function [3]. Both are also utilized in retinal development and lack of adequate amounts could adversely impact eye health. The main objective of this review is to critically evaluate the roles of choline and DHA in infant and maternal nutrition, to elucidate possible synergistic implications of both nutrients in brain and eye health and increase the awareness of this public health concern.

## 2. Role of Choline in Maternal and Infant Nutrition 

### 2.1. Essential Functions

Discovered as a component of lecithin from heated pig and ox bile in the 1860s, choline was named from the Greek word for bile, “chole” [13]. Lecithin was later characterized as being largely comprised of phosphatidylcholine, a major constituent of biological membranes. As researchers began to study how nerves communicated with each other in the 1920s, a substance secreted from a stimulated vagus nerve was identified (acetylcholine) and found to be the same molecule as a choline-containing molecule derived from fungi that could activate nerves when applied to organs. This discovery was awarded a Nobel Prize to Otto Loewi and Henry Dale in 1936.

For decades following this work, choline was recognized as being a critical component of the two biologically relevant molecules, phosphatidylcholine and acetylcholine, and yet its requirement in the human diet was substantially underappreciated. In animal models, pancreatectomized dogs developed fatty degeneration of the liver, which could be reversed by feeding raw pancreas or lecithin [14], a consequence later understood to be due to the impaired lipid packaging into lipoproteins for export from the liver. Choline deficiencies were documented in a number of other animal species, including perosis in poultry (deforming leg weakness), atherosclerosis in rodents, and related conditions in rabbit, cattle, and baboon. In addition, choline-deficient animals were found to develop liver cancer, which uncovered yet another a role of choline, as a betaine precursor in the methylation process and DNA repair [15]. Supporting these observations in animal species, human patients fed total parenteral nutrition solutions lacking choline had low circulating levels of choline, coupled with impaired liver function and fatty liver [16,17,18].

Today, choline is recognized by the National Academy of Medicine (formerly the Institute of Medicine) as an essential nutrient that must be acquired from the diet, as de novo biosynthesis is insufficient to meet human requirements [9]. We now know that choline plays a key role in lipid transport (as phosphatidylcholine in lipoprotein assembly and secretion), cell membrane structural support (as phosphatidylcholine and sphingomyelin), neurotransmission (as acetylcholine), and as a source of methyl groups (as a precursor of betaine).

### 2.2. Choline: Role in Maternal Health

In addition to the role of choline in general physiology, choline is critically important during pregnancy. Lower serum choline levels are associated with increased risk of neural tube defects [19], suggesting that choline intakes should be increased prior to pregnancy, as closing of the neural tube occurs by the fourth week of pregnancy (28 days after conception) [20]. As pregnancy progresses, the demand for choline significantly increases, as membrane biosynthesis is needed for placental development, increased workload by maternal organs, and to support exponential fetal organ growth. The growing need for choline by the developing fetus is facilitated by elevated maternal plasma free choline, placental accumulation (approximately 50 times higher concentration than maternal blood), and elevated plasma free choline levels that are three times higher than adult concentrations. Consequences of low choline status during pregnancy have major implications not only for fetal development, but also for maternal health. Acute fatty liver of pregnancy, while uncommon, is a potentially fatal complication that occurs in the third trimester or early postpartum period and shares symptoms with more common conditions like pre-eclampsia and cholestasis [21]. Furthermore, increased choline supplementation during pregnancy has demonstrated downregulation of antiangiogenic factor and preeclampsia risk marker, fms-like tyrosine kinase-1 (sFLT1) in in human placental tissues [22]. Simulating decreased placental efficiency in preeclampsia and intrauterine growth restriction, the *Dlx*3+/− mouse model has been used to show that dietary choline drives placental betaine concentration, which downregulates inflammatory gene expression [23] and improves fetal growth patterns [24].

Common genetic variants in choline-metabolizing enzymes are known to alter the metabolic fate of dietary choline, thereby increasing choline intake requirements in some individuals [25]. For instance, choline contributes to phosphatidylcholine synthesis by way of two metabolic pathways—through phosphorylation via the cytidine diphosphate-choline (CDP-choline) pathway or sequential methylation of phosphatidylethanolamine by the phosphatidylethanolamine N-methyltransferase (PEMT) pathway. The latter pathway is critically important in that it enables phosphatidylcholine synthesis even in the absence of dietary choline intake. Activity of PEMT is greater in pre-menopausal women, due to its upregulation by estrogen, but is considered inadequate to support endogenous production alone, necessitating a dietary choline requirement to prevent deficiency. Variations in single-base pairs (single-nucleotide polymorphisms or SNPs) in the *Pemt* gene are known to impact endogenous choline production, thereby influencing interindividual choline requirements and risk for choline deficiency [26,27].

Despite choline’s importance in maternal health, many do not meet the daily recommendation. Approximately 90–95% of pregnant women are consuming less than the adequate intake (AI) [28]. Experts note that choline is not found in most prenatal supplements [29,30], suggesting that increased awareness of choline’s place in infant and maternal health is needed to improve intakes through consumption of choline-rich foods and/or dietary supplementation.

### 2.3. Choline: Role in Infant Health

In the late 1970s, Steven Zeisel [31], a pediatric resident and graduate student, observed that blood choline concentrations were much higher in human newborns than in adults. Collaborator, Jan Blusztajn [32], observed that endogenous synthesis of phosphatidylcholine was increased in the brain and liver of rodent pups, demonstrating an increased metabolic demand for choline in newborns. In addition, mechanistic studies in rodents also showed that hepatic choline pools are depleted during pregnancy when fed a normal diet [33,34,35]. Soon after, choline was found to concentrate across the placenta, raising fetal tissue concentrations of choline [36]. Zeisel’s research group and colleagues subsequently discovered that mammary glands accumulate and export choline in milk, providing substantial dietary choline to the developing infant postpartum [37]. Adequate intake recommendations (AI) are 125 mg choline/day for infants 0–6 months and 150 mg choline/day for infants 7–12 months; these AIs are based on choline concentrations in human milk (160 mg/L) and mean volume output of human milk (0.78 L/day) for infants 0–6 months with a reference body weight of 7 kg (approximately 18 mg/kg), and extrapolation for reference body weight at ages 7–12 months [9].

The increased availability of choline for the brain led to the hypothesis that acetylcholine concentrations impact neurological processes and cognitive function. Testing this hypothesis, researchers Christina Williams and Warren Meck [38] found that prenatal and postnatal choline supplementation of developing rat pups improved performance as adults on the 12- and 18-arm radial maze task, a test of spatial memory. Several potential roles that maternal dietary choline play on fetal brain biochemistry that impart lifelong behavioral changes have been suggested. Amongst this is the observation that choline alters timing of neuronal differentiation in the septum and the hippocampus, two brain regions known to be involved in learning and memory [39,40]. Others have confirmed these findings, showing that prenatal choline modulated rates of adult hippocampal neurogenesis, increased hippocampal brain-derived neurotrophic factor, improved cognitive performance on memory tasks as adults [41,42], accelerated hippocampal maturation [43], and decreased the threshold for induction of long-term potentiation (an indication of synapse strength) [44,45]. These findings are especially significant, given that rates of hippocampal neurogenesis decline with age and that nutritional intervention produced a marked change in behavioral output. These impacts of dietary choline on neurogenesis extend beyond the hippocampal development, showing increased neurogenesis in the cerebral cortex [46,47] and increased levels of nerve growth factor (NGF) in both the hippocampus and cortical areas [48]. This is further supported by the observation that maternal choline status influences brain gray and white matter development in piglet brains using magnetic resonance spectroscopy neuroimaging, which is consistent with choline’s role in myelin components, phospholipids, and sphingomyelin, and emphasizes the role of choline in early brain development [49]. In humans, the role of choline in cognition have also been observed, with higher choline intakes during pregnancy associated with higher performance on memory tasks in their 7-year-old children [50]. As in brain development, emerging research by Zeisel and colleagues [51] suggests that fetal eye development may also be dependent on choline intakes, with low maternal choline intakes resulting in fewer retinal stem cells and worse eyesight in animal models. Together, the accumulating evidence underscores the long-term significance of choline in prenatal nutrition.

Beyond prenatal brain development, choline plays a significant role in continued growth of the brain and in cognitive measures. In animal models, inadequate choline intakes have demonstrated reductions in maternal plasma and milk concentrations of choline metabolites, and therefore nutrient availability for neonatal development [52]. Observational studies have shown that higher choline intakes during pregnancy have been associated with modestly better visual memory in children at age seven [50]. In a sample of 15-year-old children (*n* = 324), the sum of school grades in 17 major subjects were used as an outcome measure for academic achievement. Researchers showed that plasma choline level was significantly and positively associated with academic achievement, independent of socioeconomic status (SES) factors, and folate intake [53]. In randomized, double-blind, controlled feeding studies, maternal choline supplementation did not improve measures of infant memory or language development [54], but did improve infant information processing speed [55], bolstering support for prenatal choline supplementation. Recent evidence suggests that postnatal choline supplementation should also be considered, especially in preterm infants, when a choline deficiency may contribute to impaired lean body mass growth and neurocognitive development, despite adequate macronutrient intake and weight gain [56].

## 3. Role of DHA in Maternal and Infant Nutrition 

### 3.1. Essential Functions

While proteins and carbohydrates were widely regarded as indispensable dietary constituents by the early 20th century, there were still conflicting views on the role of dietary fats in nutrition with initial findings on essential fatty acids not emerging until the late 1920s and early 1930s [57]. Between 1929 and 1932, George O. Burr and colleagues [57,58,59,60] conducted animal studies which demonstrated that exclusion of dietary fat led to a deficiency disease in rats. Results from Burr’s work also showed that dietary fat, in particular fatty acids, was required to stimulate growth and prevent disease leading Burr to be the first to propose LA as an essential fatty acid in 1930 [58,59]. In 1931, Wesson and Burr also noted that similarly to LA, ALA is not synthesized by rats and can stimulate weight gain in rats consuming an essential fatty acid-deficient diet, which led them to conclude that ALA is also an essential fatty acid [61]. Animal work continued until 1960s to elucidate physiological actions of ALA and LA, including roles as precursors for ARA and DHA fatty acids. Key findings in 1950s led to purification of DHA and determination of its structure from animal brain phosphatides. In 1960, Klerk et al. [62] determined the metabolic pathway for conversion of DHA from ALA. Subsequent work in the 1960s and 1970s illustrated a functional role of DHA in brain and eye health, as DHA was shown to be abundant in both retinal [63,64] and synaptic membrane [65] phospholipids. Clinical research in the 1970s and 80s provided definitive findings on ALA and LA as essential fatty acids for human nutrition. Case reports and clinical studies of infants with essential fatty acid deficiency showed that it is resolved with administration of LA and/or ALA [66,67,68,69,70]. Presently, the National Academy of Medicine (formerly the Institute of Medicine) recognizes LA and ALA as essential fatty acids, recommends amounts for adequate daily intakes (AI), and state the main role of omega-3 fatty acids as structural membrane lipids, particularly in nerve tissue and retina [6]. After establishment of LA and ALA as essential fatty acids, majority of fatty acid research focused on physiological roles of common omega-3s (DHA and EPA) and omega-6 (ARA) synthesized from ALA and LA precursors, respectively. Specifically, observational findings in the 1980s and 1990s on EPA content in plasma phospholipids as well as fish oil/fish consumption (major EPA and DHA) dietary source and associations with heart health parameters led to greater understanding of the role of omega-3s in vascular biology and heart health [57]. Concurrently, omega-6 research focused on the role of ARA in eicosanoid production and downstream impact on inflammation and immune system, rather than its abundance and role in brain gray matter phospholipids. Beginning in the 1990s, focus shifted from omega-3s and heart health to role of DHA specifically in brain and eye health due to its presence in brain and retinal tissues. It is prevalent in structures that underlie cognitive abilities, including the eyes and brain (cerebellum, frontal, and occipital lobe). 

The brain is largely comprised of lipids. The predominant LC-PUFAs in the brain are ARA omega-6 fatty acid and DHA omega-3 fatty acid [5,71,72]. Both ARA and DHA can be provided in diet or synthesized via desaturation and elongation pathways from LA and ALA precursors. Findings show DHA affects brain function by modulating neurogenesis [73,74], influencing neurotransmission [75], and promoting synaptic activity [76]. Additionally, DHA exerts other important actions that affect overall health including playing a key role in cell signaling, lipid metabolism, cell membrane function, and eye development and function. Docosahexaenoic acid (DHA) acts as a precursor for autocoid signaling molecules as well as an activator of several gene transcription factors including peroxisome proliferator activated receptors [77], which modulate metabolic processes, cholesterol homeostasis, and inflammation [78]. It also impacts cell membrane properties by increasing membrane permeability [79,80,81,82,83] and facilitating membrane fluidity [84,85,86,87]. Docosahexaenoic acid contributes to eye development and function through its signaling [88] and structural [89] effects on retinal photoreceptor cells. Low levels of plasma DHA have been observed in individuals with genetic retinal disorders such as retinitis pigmentosa leading some to theorize that DHA deficiencies could lead to defects in central retinal cones adversely impacting eye health [90]. In all, the totality of scientific literature strongly suggests a role of dietary DHA in various aspects of health including neonatal neurodevelopment. 

### 3.2. DHA: Role in Maternal Health 

Pregnancy is a time when higher nutrient demands can impact maternal health and pregnancy outcomes. Maternal DHA intake, endogenous synthesis, and body stores help meet DHA needs for mother and fetus through pregnancy [86,91]. Various genetic and lifestyle factors can influence maternal DHA stores including nutrition as well as pre-pregnancy health conditions, which may alter DHA utilization. Impaired fatty acid metabolism, due to the various single nucleotide polymorphisms (SNPs), can affect DHA status, and consequently, alter DHA requirements [92]. Researchers assessed several SNPs for genes encoding for key enzymes involved in LC-PUFA production—fatty acid desaturase (FADS) and elongase (ELOVL)—and found that *FADS1* and *FADS2* variants impacted mainly omega-6 fatty acid levels, whereas *ELOVL* genetic variants affected omega-3 fatty acid levels [93]. Investigators also observed normal weight women with *FADS1* SNPs had significantly lower levels of ARA and overweight/obese women with ELOVL2 SNPs had an association with lower DHA levels [93]. Gonzalez-Casanova et al. [94] examined the impact of 15 different FADS SNPs along with prenatal DHA supplementation on birth weight in the Mexican population based on earlier observations of high prevalence of SNPs associated with slow LC-PUFA conversion in Hispanic populations. Results showed differential response to prenatal supplementation due to the FADS genotype. Women with *FADS2* SNP that received supplementation had higher birth weight children than those who received the placebo. This led the authors to propose that mixed findings on DHA supplementation’s effects on birth weight could be attributed to differential responses due to genetic variance [94]. Xie and Innis [95] found that genetic variation in *FADS1* and *FADS2* influenced maternal plasma and erythrocyte levels of both omega-3 (DHA) and omega-6 (ARA) fatty acids during pregnancy and lipids levels in breast milk during lactation. Overall maternal health status can also impact the role of DHA during pregnancy. A recent study showed pre-pregnancy obesity is associated with higher inflammation and attenuated response to DHA supplementation [96]. In general, the DHA needs of the fetus rise during pregnancy, peaking towards the end of the third trimester (after week 32), a time of great growth and development as well as significant accretion of DHA in fetal brain tissue [97,98,99,100]. Kuipers et al. [100] evaluated DHA fetal content at 25, 35, and 40 weeks of gestation. At term (40 weeks), 21% of DHA was present in skeletal muscle, 23% in brain tissue, and most of the DHA residing in fetal adipose tissue (50%), leading researchers to suggest fetal DHA adipose content serves as a reservoir for DHA that can be utilized in infant development. Higher maternal DHA intakes may be required in the late stage of pregnancy to help meet elevating needs. With advancing gestation, there is increased transfer of DHA to the developing fetus which may lead to depleted maternal DHA levels [101]. Low levels of DHA have been associated with increased incidence of various diseases during perinatal period, including postpartum depression [101]. Maternal DHA supplementation (600 mg daily) starting in the last half of gestation (week 20 to birth) resulted in greater gestational duration and infant birth size [102]. Observational and clinical research shows maternal DHA dietary intakes during pregnancy impact blood DHA status of newborns [103]. Maternal and fetal DHA is associated with several brain and eye health outcomes in infants [77,103,104].

### 3.3. DHA: Role in Infant Health

Maternal DHA status during fetal development can have lasting impacts on brain and eye health throughout life as evident by key recommendations for DHA intakes during the first 1000 days of life [3]. Docosahexaenoic acid is essential for normal vision and brain development. There is rapid DHA accretion in utero during the last weeks of the third trimester to support growth [77,97,98,99,100,105,106] and DHA is a major structural lipid in both the brain and retina. It reaches levels of about 4 g in the brain and represents 50% of the fatty acids present in retinal rod and cone components [106]. During pregnancy, DHA insufficiency was noticed to be associated with lower infant neurodevelopment scores up to 18 months old; however, this finding was not detected at later follow up (5 years of age) [107]. Maternal DHA intake, particularly in the last 5 weeks of pregnancy, influences visual acuity development up towards the end of the first year of life [108]. 

Recent studies have observed an effect of maternal and fetal DHA status on general health including various brain and eye health outcomes well into early and mid-childhood. Researchers found associations with umbilical cord plasma DHA levels and metabolic measures in early childhood (3 years of age) [109]. Specifically, higher cord plasma DHA was associated with lower body mass index (BMI) scores, waist circumference, and leptin levels. This association was stronger when infant–mother pairs experienced hyperglycemia during pregnancy [109]. Prenatal DHA status may also contribute to improved sustained attention in preschool children [110]. In contrast, some findings have not shown an impact of maternal DHA on brain health measures in childhood. Crozier et al. [111] found neither maternal ARA or DHA levels during pregnancy were associated with cognitive performance in 4- and 6-year-old children. Investigators speculated that diet quality, which was positively associated with neurodevelopment at four years of age may compensate for maternal DHA insufficiencies during pregnancy [111], indicating an effect of postnatal nutrition on health as well. Daily postnatal DHA supplementation, via DHA enriched solid baby food from 6–12 months old, led to improved visual acuity [112]. Some early communicative development benefits were also observed with DHA supplementation during early infancy [113]. However, other neurodevelopment outcomes were not significantly changed in the study [113]. Current findings indicate various factors affect the overall impact of DHA in infant brain and eye health including both pre-pregnancy maternal health status as well as prenatal and postnatal nutritional status of mother and child. 

## 4. Synergistic Relationship between Choline and DHA 

### 4.1. Proposed Mechanisms: Insights from Preclinical Studies 

Choline and DHA are two important lipid-based nutrients needed for growth and development. Findings from in vitro studies highlight potential interactions between both nutrients and effects on metabolism. For example, DHA supplementation significantly increased cellular choline uptake in human retinal cells compared to cultured cells without supplemental DHA [114], which demonstrates how DHA may impact choline status. This finding is consistent with an earlier report demonstrating that the high unsaturated fatty acid content of human retinoblastoma cell membranes facilitated the capacity of the high-affinity choline uptake system to transport low concentrations of choline [114,115]. Docosahexaenoic acid has been shown to stimulate choline acetyltransferase (ChAT) enzymatic activity as well as support growth and function in neural cell culture studies [116,117]. The interaction of DHA and choline status has also been demonstrated in vivo, in the *Pemt−/−* mouse model. Discussed in Section 2.2, the PEMT enzyme facilitates endogenous hepatic synthesis of phosphatidylcholine from phosphatidylethanolamine. Deficiency of PEMT in *Pemt* gene knockout (*Pemt−/−*) mice yields disrupted fetal hippocampal development that can be reversed through maternal dietary DHA supplementation compared to control diets [118]. Maternal DHA supplementation not only increased fetal brain *Pemt−/−* phospholipid-DHA levels to wild-type levels, and eliminated differences in neural progenitor cell proliferation and apoptosis differences, but the DHA supplementation also halved the rate of neural apoptosis in wild type [118], affirming roles of DHA and choline in developmental neurogenesis. This observation is consistent with the specificity of PEMT in synthesizing polyunsaturated-phosphatidylcholine species [119] and highlights the coordinated roles of choline and DHA in neural lipid metabolism. Recent work in evaluating aspects of neural development processes revealed that specific nutrient combinations have a beneficial impact on brain function. Both in vitro and in vivo studies illustrate the role of nutrient combinations in neurodevelopment, particularly the presence of both DHA and choline. van Deijk et al. [120] showed that a medical food with DHA and choline, as well as other micronutrients, positively affected synaptic function in neuronal cells. When assessing aspects of neuroinflammation, investigators observed that administration of a DHA-containing choline phospholipid led to decreased lipopolysaccharide (LPS)-induced neuroinflammation in both cell culture and animal models [121]. Similar to its anti-inflammatory effects, supplementing with choline and DHA also attenuated brain oxidative stress in a perinatal maternal separation stress rodent model [122]. Favorable effects of nutrient combinations were also seen when supplementing during pregnancy. Results from several studies indicate a valuable impact of prenatal choline and DHA supplementation on offspring brain function in rats [123,124], dogs [125], and pigs [126]. In a study of choline, DHA, or saline control supplementation of pregnant rat dams and pups from choline or DHA supplemented groups showed significantly increased numbers of CA1 hippocampal neurons compared to both untreated and saline control group pups (*p* < 0.05). The combined supplementation of choline and DHA during pregnancy further enhanced neurodevelopment of the fetal hippocampus in rats compared to control (*p* < 0.001), with these effects proving better than supplementing with choline (*p* < 0.05) or DHA (*p* < 0.05) alone [124]. These results are supported by work by van Wijk et al. [123], who showed that supplementation of DHA in rats significantly increased DHA levels in plasma and red blood cells compared to control, and supplementation of choline from lecithin significantly increased plasma free choline, but did not affect DHA levels. However, combined supplementation of DHA and lecithin increased DHA to levels significantly greater than DHA supplementation alone (*p* < 0.025), which indicated that the impact of combined choline and DHA supplementation on circulating DHA levels were greater than an additive effect [123]. Advantageous effects of nutrient combinations could be due to the role of choline in placental nutrient transport. Kwan et al. [127] found that maternal choline supplementation modulates placental nutrient metabolism in a late gestational mouse pregnancy model, which may be attributed to its influence on vascular development. Furthermore, maternal choline supplementation increased placental transcript abundance of DHA, choline, and acetylcholine transporters [127]. Collectively, the evidence presented in this section supports the hypothesis that supplementation of choline or DHA alone yield complementary outcomes on choline and DHA metabolism, and that supplementation in combination produce benefits that must be greater than additive effects. Current in vivo and in vitro evidence supports co-supplementation of choline and DHA, particularly during pregnancy to support brain and eye development of offspring, and while the literature to date provides novel leads on how choline and DHA function in synergy, additional work is needed to clarify its mechanism of action. 

Choline and DHA are two important lipid-based nutrients needed for growth and development. Findings from in vitro studies highlight potential interactions between both nutrients and effects on metabolism. For example, DHA supplementation significantly increased cellular choline uptake in human retinal cells compared to cultured cells without supplemental DHA [114], which demonstrates how DHA may impact choline status. This finding is consistent with an earlier report demonstrating that the high unsaturated fatty acid content of human retinoblastoma cell membranes facilitated the capacity of the high-affinity choline uptake system to transport low concentrations of choline [114,115]. Docosahexaenoic acid has been shown to stimulate choline acetyltransferase (ChAT) enzymatic activity as well as support growth and function in neural cell culture studies [116,117]. The interaction of DHA and choline status has also been demonstrated in vivo, in the *Pemt−/−* mouse model. Discussed in Section 2.2, the PEMT enzyme facilitates endogenous hepatic synthesis of phosphatidylcholine from phosphatidylethanolamine. Deficiency of PEMT in *Pemt* gene knockout (*Pemt−/−*) mice yields disrupted fetal hippocampal development that can be reversed through maternal dietary DHA supplementation compared to control diets [118]. Maternal DHA supplementation not only increased fetal brain *Pemt−/−* phospholipid-DHA levels to wild-type levels, and eliminated differences in neural progenitor cell proliferation and apoptosis differences, but the DHA supplementation also halved the rate of neural apoptosis in wild type [118], affirming roles of DHA and choline in developmental neurogenesis. This observation is consistent with the specificity of PEMT in synthesizing polyunsaturated-phosphatidylcholine species [119] and highlights the coordinated roles of choline and DHA in neural lipid metabolism. Recent work in evaluating aspects of neural development processes revealed that specific nutrient combinations have a beneficial impact on brain function. Both in vitro and in vivo studies illustrate the role of nutrient combinations in neurodevelopment, particularly the presence of both DHA and choline. van Deijk et al. [120] showed that a medical food with DHA and choline, as well as other micronutrients, positively affected synaptic function in neuronal cells. When assessing aspects of neuroinflammation, investigators observed that administration of a DHA-containing choline phospholipid led to decreased lipopolysaccharide (LPS)-induced neuroinflammation in both cell culture and animal models [121]. Similar to its anti-inflammatory effects, supplementing with choline and DHA also attenuated brain oxidative stress in a perinatal maternal separation stress rodent model [122]. Favorable effects of nutrient combinations were also seen when supplementing during pregnancy. Results from several studies indicate a valuable impact of prenatal choline and DHA supplementation on offspring brain function in rats [123,124], dogs [125], and pigs [126]. In a study of choline, DHA, or saline control supplementation of pregnant rat dams and pups from choline or DHA supplemented groups showed significantly increased numbers of CA1 hippocampal neurons compared to both untreated and saline control group pups (*p* < 0.05). The combined supplementation of choline and DHA during pregnancy further enhanced neurodevelopment of the fetal hippocampus in rats compared to control (*p* < 0.001), with these effects proving better than supplementing with choline (*p* < 0.05) or DHA (*p* < 0.05) alone [124]. These results are supported by work by van Wijk et al. [123], who showed that supplementation of DHA in rats significantly increased DHA levels in plasma and red blood cells compared to control, and supplementation of choline from lecithin significantly increased plasma free choline, but did not affect DHA levels. However, combined supplementation of DHA and lecithin increased DHA to levels significantly greater than DHA supplementation alone (*p* < 0.025), which indicated that the impact of combined choline and DHA supplementation on circulating DHA levels were greater than an additive effect [123]. Advantageous effects of nutrient combinations could be due to the role of choline in placental nutrient transport. Kwan et al. [127] found that maternal choline supplementation modulates placental nutrient metabolism in a late gestational mouse pregnancy model, which may be attributed to its influence on vascular development. Furthermore, maternal choline supplementation increased placental transcript abundance of DHA, choline, and acetylcholine transporters [127]. Collectively, the evidence presented in this section supports the hypothesis that supplementation of choline or DHA alone yield complementary outcomes on choline and DHA metabolism, and that supplementation in combination produce benefits that must be greater than additive effects. Current in vivo and in vitro evidence supports co-supplementation of choline and DHA, particularly during pregnancy to support brain and eye development of offspring, and while the literature to date provides novel leads on how choline and DHA function in synergy, additional work is needed to clarify its mechanism of action. 

### 4.2. Proposed Mechanisms: Insights from Clinical Findings

In this review, we have thus far discussed the roles of choline and DHA independently on infant brain and eye development, and synergistic relationships from preclinical models. From a clinical standpoint, the roles of choline and DHA on infant development may be best observed in premature birth, a circumstance wherein nutrient availability is interrupted, when levels would otherwise remain high *in utero*. Furthermore, human brain and eye development is known to begin *in utero*, and continue postnatally, with choline and LC-PUFAs (DHA, ARA) supplied together postpartum from human milk or supplemental infant formula. Human milk choline content from mothers of preterm infants have also been reported to be lower than from mothers following full-term delivery [128]. Preterm births in the US occurred in about 1 in 10 babies from 2007 to 2014, with an estimated associated cost of $26.2 billion USD annually, equating to approximately $51,600 per infant born preterm [129,130]. More importantly, premature births are associated with increased health problems, longer hospital stays, and learning and behavioral problems through childhood. To highlight the mechanistic synergy of choline and DHA, Bernhard and colleagues [131] demonstrated through a randomized, controlled trial that in preterm infants supplemented with choline, DHA, both, or neither, choline alone did not significantly increase phosphatidylcholine-DHA (PC-DHA), DHA alone increased PC-DHA content by only 35% (*p* < 0.05), but the combined treatment increased PC-DHA by 63% compared to control (*p* < 0.001). Additionally, supplementation with choline increased plasma choline concentrations in preterm infants to near-fetal concentrations and improved DHA status [131]. Since phosphatidylcholine is the major transport form of DHA in plasma, these findings provide strong support for the hypothesis that actions of each nutrient are metabolically linked through phosphatidylcholine and that their actions cannot be mutually exclusive.

To better understand the role(s) choline and DHA play in unison in infant brain and eye health, researchers have studied how choline intakes influence phosphatidylcholine-DHA enrichment [132]. West and colleagues [133] showed that phosphatidylcholine-DHA levels were greater in pregnant women than non-pregnant women, suggesting a greater demand for methyl donors and increased PEMT activity. Additionally, they showed that when choline and DHA were both supplemented in non-pregnant women, higher choline intakes resulted in greater PC-DHA enrichment, suggesting that higher choline intakes may increase PEMT activity [132,134]. These findings suggest a metabolic synergy between choline and DHA, and are further supported by research that demonstrated consumption of n-3 fatty acid-enriched eggs increased plasma free choline and betaine compared to non-enriched eggs [133]. Consistent with these findings, McNamara et al. [135] showed that low DHA status is associated with lower cortical metabolism of choline using proton magnetic resonance spectroscopy. To explore the relationship between infant nutrition and subsequent cognitive performance, Cheatham et al. [136] collected and analyzed human milk samples at 3–4 months postpartum and tested recognition memory ability in infants at 6-months old. Through this work, Cheatham demonstrated that brain electrical activity expressed as event-related potential latency scores from the frontal, central, and midline areas were predicted by the DHA and choline interaction and that higher choline and DHA content was related to better recognition memory [136]. Together, these clinical findings provide evidence that choline and DHA function is synergistic in infant neurocognitive development.

## 5. Conclusions and Future Directions

The evidence presented in this review of cell culture, animal model, and human clinical trials provide compelling support for choline and DHA in maternal and infant nutrition. The interactions between choline and DHA support roles in brain and eye health, and the findings that supplementation with one augments the other points to synergy between the two nutrients. Dietary intakes of choline and DHA in the US population are lower than recommended levels, and vulnerable populations may require additional supplementation, particularly women during pregnancy and lactation. Infants receiving nutrition from breastmilk are provided with choline and LC-PUFAs (inclusive of ARA, LA, and DHA) from the maternal intake, but those who are not breastfed will require formula that contains choline and LC-PUFAs for proper growth and development.

Ensuring optimal nutrition during pregnancy requires a multi-faceted approach. Effective strategies are needed to determine nutrient status, address key nutrient gaps, and gain a deeper understanding of nutrient interactions during pregnancy. Prenatal testing for DHA status has been proposed [137] and should also include choline to inform on nutrient adequacy and guide strategy for obtaining optimal intake levels. Moreover, commercial tools for screening of common SNPs may enable personalized tailoring of dietary recommendations. With a greater understanding of maternal nutrient needs and how genetic and lifestyle factors impact metabolism, we are empowered to improve nutritional approaches to prenatal care.

## References

[1] Rui O.T.P. National Ambulatory Medical Care Survey: 2015 State and National Summary Tables. https://www.cdc.gov/nchs/data/ahcd/namcs_summary/2015_namcs_web_tables.pdf.

[2] US Department of Health and Human Services Healthy People 2020: Maternal, Infant, and Child Health Objectives. https://www.healthypeople.gov/2020/topics-objectives/topic/maternal-infant-and-child-health/objectives.

[3] Schwarzenberg S.J., Georgieff M.K., Committee on Nutrition (2018). Advocacy for Improving Nutrition in the First 1000 Days to Support Childhood Development and Adult Health. Pediatrics.

[4] Koletzko B., Carlson S.E., van Goudoever J.B. (2015). Should Infant Formula Provide Both Omega-3 DHA and Omega-6 Arachidonic Acid?. Ann. Nutr. Metab..

[5] Martinez M. (1992). Tissue levels of polyunsaturated fatty acids during early human development. J. Pediatr..

[6] Institute of Medicine (US) (2006). Dietary Fat: Total Fat and Fatty Acids. Dietary References Intakes: The Essential Guide to Nutrient Requirements.

[7] US Department of Agriculture—Agricultural Research Service Nutrient Intakes from Food: Mean Amounts Consumed per Individual, by Gender and Age. https://www.ars.usda.gov/ARSUserFiles/80400530/pdf/1516/Table_1_NIN_GEN_15.pdf.

[8] Wallace T.C., Fulgoni V.L. (2016). Assessment of Total Choline Intakes in the United States. J. Am. Coll. Nutr..

[9] IoM (US) (1998). Choline. Dietary Reference Intakes for Thiamin, Riboflavin, Niacin, Vitamin B_6_, Folate, Vitamin B_12_, Pantothenic Acid, Biotin, and Choline.

[10] Papanikolaou Y., Brooks J., Reider C., Fulgoni V.L. (2014). US adults are not meeting recommended levels for fish and omega-3 fatty acid intake: Results of an analysis using observational data from NHANES 2003–2008. Nutr. J..

[11] US Department of Health and Human Services 2015–2020 Dietary Guidelines for Americans. https://health.gov/dietaryguidelines/2015/resources/2015-2020_Dietary_Guidelines.pdf.

[12] Zhang Z., Fulgoni V.L., Kris-Etherton P.M., Mitmesser S.H. (2018). Dietary Intakes of EPA and DHA Omega-3 Fatty Acids among US Childbearing-Age and Pregnant Women: An Analysis of NHANES 2001–2014. Nutrients.

[13] Zeisel S.H. (2012). A brief history of choline. Ann. Nutr. Metab..

[14] Hershey J.M., Soskin S. (1931). Substitution of “lecithin” for raw pancreas in the diet of the depancreatized dog. Am. J. Physiol.-Leg. Content.

[15] Mehedint M.G., Zeisel S.H. (2013). Choline’s role in maintaining liver function: New evidence for epigenetic mechanisms. Curr. Opin. Clin. Nutr. Metab. Care.

[16] Chawla R.K., Berry C.J., Kutner M.H., Rudman D. (1985). Plasma concentrations of transsulfuration pathway products during nasoenteral and intravenous hyperalimentation of malnourished patients. Am. J. Clin. Nutr..

[17] Sheard N.F., Tayek J.A., Bistrian B.R., Blackburn G.L., Zeisel S.H. (1986). Plasma choline concentration in humans fed parenterally. Am. J. Clin. Nutr..

[18] Tayek J.A., Bistrian B., Sheard N.F., Zeisel S.H., Blackburn G.L. (1990). Abnormal liver function in malnourished patients receiving total parenteral nutrition: A prospective randomized study. J. Am. Coll. Nutr..

[19] Shaw G.M., Finnell R.H., Blom H.J., Carmichael S.L., Vollset S.E., Yang W., Ueland P.M. (2009). Choline and risk of neural tube defects in a folate-fortified population. Epidemiology.

[20] Cavalli P. (2008). Prevention of Neural Tube Defects and proper folate periconceptional supplementation. J. Prenat. Med..

[21] Ko H., Yoshida E.M. (2006). Acute fatty liver of pregnancy. Can. J. Gastroenterol. J. Can. Gastroenterol..

[22] Jiang X., Bar H.Y., Yan J., Jones S., Brannon P.M., West A.A., Perry C.A., Ganti A., Pressman E., Devapatla S. (2013). A higher maternal choline intake among third-trimester pregnant women lowers placental and circulating concentrations of the antiangiogenic factor fms-like tyrosine kinase-1 (sFLT1). FASEB J..

[23] King J.H., Kwan S.T., Yan J., Jiang X., Fomin V.G., Roberson M.S., Caudill M.A. (2016). Maternal Choline Supplementation Modulates Maternal and Fetal Choline Metabolism and Downregulates Inflammatory Gene Expression in a Mouse Model of Placental Insufficiency. FASEB J..

[24] King J.H., Kwan S.T.C., Yan J., Klatt K.C., Jiang X., Roberson M.S., Caudill M.A. (2017). Maternal Choline Supplementation Alters Fetal Growth Patterns in a Mouse Model of Placental Insufficiency. Nutrients.

[25] Ganz A.B., Cohen V.V., Swersky C.C., Stover J., Vitiello G.A., Lovesky J., Chuang J.C., Shields K., Fomin V.G., Lopez Y.S. (2017). Genetic Variation in Choline-Metabolizing Enzymes Alters Choline Metabolism in Young Women Consuming Choline Intakes Meeting Current Recommendations. Int. J. Mol. Sci..

[26] da Costa K.A., Kozyreva O.G., Song J., Galanko J.A., Fischer L.M., Zeisel S.H. (2006). Common genetic polymorphisms affect the human requirement for the nutrient choline. FASEB J..

[27] Resseguie M.E., da Costa K.-A., Galanko J.A., Patel M., Davis I.J., Zeisel S.H. (2011). Aberrant Estrogen Regulation of PEMT Results in Choline Deficiency-associated Liver Dysfunction. J. Biol. Chem..

[28] Brunst K.J., Wright R.O., DiGioia K., Enlow M.B., Fernandez H., Wright R.J., Kannan S. (2014). Racial/ethnic and sociodemographic factors associated with micronutrient intakes and inadequacies among pregnant women in an urban US population. Public Health Nutr..

[29] Bell C., Aujla J. (2016). Prenatal vitamins deficient in recommended Choline intake for pregnant women. J. Fam. Med. Dis. Prev..

[30] Caudill M.A. (2010). Pre- and postnatal health: Evidence of increased choline needs. J. Am. Diet. Assoc..

[31] Zeisel S.H., Epstein M.F., Wurtman R.J. (1980). Elevated choline concentration in neonatal plasma. Life Sci..

[32] Blusztajn J.K., Zeisel S.H., Wurtman R.J. (1979). Synthesis of lecithin (phosphatidylcholine) from phosphatidylethanolamine in bovine brain. Brain Res..

[33] Gwee M.C., Sim M.K. (1978). Free choline concentration and cephalin-N-methyltransferase activity in the maternal and foetal liver and placenta of pregnant rats. Clin. Exp. Pharmacol. Physiol..

[34] Gwee M.C., Sim M.K. (1979). Changes in the concentration of free choline and cephalin-N-methyltransferase activity of the rat material and foetal liver and placeta during gestation and of the maternal and neonatal liver in the early postpartum period. Clin. Exp. Pharmacol. Physiol..

[35] Zeisel S.H., Mar M.H., Zhou Z., da Costa K.A. (1995). Pregnancy and lactation are associated with diminished concentrations of choline and its metabolites in rat liver. J. Nutr..

[36] Sweiry J.H., Page K.R., Dacke C.G., Abramovich D.R., Yudilevich D.L. (1986). Evidence of saturable uptake mechanisms at maternal and fetal sides of the perfused human placenta by rapid paired-tracer dilution: Studies with calcium and choline. J. Dev. Physiol..

[37] Chao C.K., Pomfret E.A., Zeisel S.H. (1988). Uptake of choline by rat mammary-gland epithelial cells. Biochem. J..

[38] Meck W.H., Smith R.A., Williams C.L. (1988). Pre- and postnatal choline supplementation produces long-term facilitation of spatial memory. Dev. Psychobiol..

[39] Albright C.D., Friedrich C.B., Brown E.C., Mar M.H., Zeisel S.H. (1999). Maternal dietary choline availability alters mitosis, apoptosis and the localization of TOAD-64 protein in the developing fetal rat septum. Brain Res. Dev. Brain Res..

[40] Craciunescu C.N., Albright C.D., Mar M.H., Song J., Zeisel S.H. (2003). Choline availability during embryonic development alters progenitor cell mitosis in developing mouse hippocampus. J. Nutr..

[41] Glenn M.J., Gibson E.M., Kirby E.D., Mellott T.J., Blusztajn J.K., Williams C.L. (2007). Prenatal choline availability modulates hippocampal neurogenesis and neurogenic responses to enriching experiences in adult female rats. Eur. J. Neurosci..

[42] Tees R.C., Mohammadi E. (1999). The effects of neonatal choline dietary supplementation on adult spatial and configural learning and memory in rats. Dev. Psychobiol..

[43] Mellott T.J., Williams C.L., Meck W.H., Blusztajn J.K. (2004). Prenatal choline supplementation advances hippocampal development and enhances MAPK and CREB activation. FASEB J..

[44] Pyapali G.K., Turner D.A., Williams C.L., Meck W.H., Swartzwelder H.S. (1998). Prenatal dietary choline supplementation decreases the threshold for induction of long-term potentiation in young adult rats. J. Neurophysiol..

[45] Jones J.P., Meck W.H., Williams C.L., Wilson W.A., Swartzwelder H.S. (1999). Choline availability to the developing rat fetus alters adult hippocampal long-term potentiation. Brain Res. Dev. Brain Res..

[46] Trujillo-Gonzalez I., Wang Y., Friday W.B., Vickers K.C., Toth C.L., Molina-Torres L., Surzenko N., Zeisel S.H. (2018). microRNA-129-5p is regulated by choline availability and controls EGF receptor synthesis and neurogenesis in the cerebral cortex. FASEB J..

[47] Wang Y., Surzenko N., Friday W.B., Zeisel S.H. (2016). Maternal dietary intake of choline in mice regulates development of the cerebral cortex in the offspring. FASEB J..

[48] Sandstrom N.J., Loy R., Williams C.L. (2002). Prenatal choline supplementation increases NGF levels in the hippocampus and frontal cortex of young and adult rats. Brain Res..

[49] Mudd A.T., Getty C.M., Dilger R.N. (2018). Maternal Dietary Choline Status Influences Brain Gray and White Matter Development in Young Pigs. Curr. Dev. Nutr..

[50] Boeke C.E., Gillman M.W., Hughes M.D., Rifas-Shiman S.L., Villamor E., Oken E. (2013). Choline intake during pregnancy and child cognition at age 7 years. Am. J. Epidemiol..

[51] Surzenko N., Trujillo-González I., Zeisel S.H. (2016). Low Intake of Choline During Pregnancy Leads to Aberrant Retinal Architecture and Poor Visual Function in the Offspring. FASEB J..

[52] Mudd A.T., Alexander L.S., Johnson S.K., Getty C.M., Malysheva O.V., Caudill M.A., Dilger R.N. (2016). Perinatal Dietary Choline Deficiency in Sows Influences Concentrations of Choline Metabolites, Fatty Acids, and Amino Acids in Milk throughout Lactation. J. Nutr..

[53] Nilsson T.K., Hurtig-Wennlof A., Sjostrom M., Herrmann W., Obeid R., Owen J.R., Zeisel S. (2016). Plasma 1-carbon metabolites and academic achievement in 15-yr-old adolescents. FASEB J..

[54] Cheatham C.L., Goldman B.D., Fischer L.M., da Costa K.A., Reznick J.S., Zeisel S.H. (2012). Phosphatidylcholine supplementation in pregnant women consuming moderate-choline diets does not enhance infant cognitive function: A randomized, double-blind, placebo-controlled trial. Am. J. Clin. Nutr..

[55] Caudill M.A., Strupp B.J., Muscalu L., Nevins J.E.H., Canfield R.L. (2018). Maternal choline supplementation during the third trimester of pregnancy improves infant information processing speed: A randomized, double-blind, controlled feeding study. FASEB J..

[56] Bernhard W., Poets C.F., Franz A.R. (2018). Choline and choline-related nutrients in regular and preterm infant growth. Eur. J. Nutr..

[57] Spector A.A., Kim H.Y. (2015). Discovery of essential fatty acids. J. Lipid Res..

[58] Burr G.O., Burr M.M. (1929). A new deficiency disease produced by the rigid exclusion of fat from the diet. J. Biol. Chem..

[59] Burr G.O., Burr M.M. (1930). On the nature and role of the fatty acids essential in nutrition. J. Biol. Chem..

[60] Burr G.O., Burr M.M., Miller E.S. (1932). On the fatty acids essential in nutrition. III. J. Biol. Chem..

[61] Wesson L.G., Burr G.O. (1931). The metabolic rate and respiratory quotients of rats on a fat-deficient diet. J. Biol. Chem..

[62] Klenk E., Mohrhauer H. (1960). Studies on the metabolism of polyenoic fatty acids in the rat. Hoppe Seylers Z. Physiol. Chem..

[63] Anderson R.E., Benolken R.M., Dudley P.A., Landis D.J., Wheeler T.G. (1974). Proceedings: Polyunsaturated fatty acids of photoreceptor membranes. Exp. Eye Res..

[64] Benolken R.M., Anderson R.E., Wheeler T.G. (1973). Membrane fatty acids associated with the electrical response in visual excitation. Science.

[65] Cotman C., Blank M.L., Moehl A., Snyder F. (1969). Lipid composition of synaptic plasma membranes isolated from rat brain by zonal centrifugation. Biochemistry.

[66] Caldwell M.D., Jonsson H.T., Othersen H.B. (1972). Essential fatty acid deficiency in an infant receiving prolonged parenteral alimentation. J. Pediatr..

[67] Collins F.D., Sinclair A.J., Royle J.P., Coats D.A., Maynard A.T., Leonard R.F. (1971). Plasma lipids in human linoleic acid deficiency. Nutr. Metab..

[68] Holman R.T., Johnson S.B., Hatch T.F. (1982). A case of human linolenic acid deficiency involving neurological abnormalities. Am. J. Clin. Nutr..

[69] Postuma R., Pease P.W., Watts R., Taylor S., McEvoy F.A. (1978). Essential fatty acid deficiency in infants receiving parenteral nutrition. J. Pediatr. Surg..

[70] Tashiro T., Ogata H., Yokoyama H., Mashima Y., Itoh K. (1976). The effect of fat emulsion (Intralipid) on essential fatty acid deficiency in infants receiving intravenous alimentation. J. Pediatr. Surg..

[71] Carlson S.E., Colombo J. (2016). Docosahexaenoic Acid and Arachidonic Acid Nutrition in Early Development. Adv. Pediatr..

[72] Svennerholm L., Vanier M.T. (1973). The distribution of lipids in the human nervous system. 3. Fatty acid composition of phosphoglycerides of human foetal and infant brain. Brain Res..

[73] Dyall S.C., Michael G.J., Michael-Titus A.T. (2010). Omega-3 fatty acids reverse age-related decreases in nuclear receptors and increase neurogenesis in old rats. J. Neurosci. Res..

[74] Janssen C.I., Zerbi V., Mutsaers M.P., de Jong B.S., Wiesmann M., Arnoldussen I.A., Geenen B., Heerschap A., Muskiet F.A., Jouni Z.E. (2015). Impact of dietary n-3 polyunsaturated fatty acids on cognition, motor skills and hippocampal neurogenesis in developing C57BL/6J mice. J. Nutr. Biochem..

[75] Kodas E., Galineau L., Bodard S., Vancassel S., Guilloteau D., Besnard J.C., Chalon S. (2004). Serotoninergic neurotransmission is affected by n-3 polyunsaturated fatty acids in the rat. J. Neurochem..

[76] Cao D., Kevala K., Kim J., Moon H.S., Jun S.B., Lovinger D., Kim H.Y. (2009). Docosahexaenoic acid promotes hippocampal neuronal development and synaptic function. J. Neurochem..

[77] Lauritzen L., Brambilla P., Mazzocchi A., Harslof L.B., Ciappolino V., Agostoni C. (2016). DHA Effects in Brain Development and Function. Nutrients.

[78] Muskiet F.A., Fokkema M.R., Schaafsma A., Boersma E.R., Crawford M.A. (2004). Is Docosahexaenoic Acid (DHA) Essential? Lessons from DHA Status Regulation, Our Ancient Diet, Epidemiology and Randomized Controlled Trials. J. Nutr..

[79] Ehringer W., Belcher D., Wassall S.R., Stillwell W. (1990). A comparison of the effects of linolenic (18:3 omega 3) and docosahexaenoic (22:6 omega 3) acids on phospholipid bilayers. Chem. Phys. Lipids.

[80] Jenski L.J., Sturdevant L.K., Ehringer W.D., Stillwell W. (1993). Omega-3 fatty acid modification of membrane structure and function. I. Dietary manipulation of tumor cell susceptibility to cell- and complement-mediated lysis. Nutr. Cancer.

[81] Stillwell W., Ehringer W., Jenski L.J. (1993). Docosahexaenoic acid increases permeability of lipid vesicles and tumor cells. Lipids.

[82] Stillwell W., Jenski L.J., Crump F.T., Ehringer W. (1997). Effect of docosahexaenoic acid on mouse mitochondrial membrane properties. Lipids.

[83] Stillwell W., Wassall S.R. (2003). Docosahexaenoic acid: Membrane properties of a unique fatty acid. Chem. Phys. Lipids.

[84] Lee A.G., East J.M., Froud R.J. (1986). Are essential fatty acids essential for membrane function?. Prog. Lipid Res..

[85] Stubbs C.D., Smith A.D. (1984). The modification of mammalian membrane polyunsaturated fatty acid composition in relation to membrane fluidity and function. Biochim. Biophys. Acta.

[86] Uauy R., Mena P., Rojas C. (2000). Essential fatty acids in early life: Structural and functional role. Proc. Nutr. Soc..

[87] Wheeler T.G., Benolken R.M., Anderson R.E. (1975). Visual membranes: Specificity of fatty acid precursors for the electrical response to illumination. Science.

[88] Knott E.J., Gordon W.C., Jun B., Do K., Bazan N.G. (2018). Retinal Pigment Epithelium and Photoreceptor Preconditioning Protection Requires Docosanoid Signaling. Cell. Mol. Neurobiol..

[89] Shindou H., Koso H., Sasaki J., Nakanishi H., Sagara H., Nakagawa K.M., Takahashi Y., Hishikawa D., Iizuka-Hishikawa Y., Tokumasu F. (2017). Docosahexaenoic acid preserves visual function by maintaining correct disc morphology in retinal photoreceptor cells. J. Biol. Chem..

[90] Gong J., Rosner B., Rees D.G., Berson E.L., Weigel-DiFranco C.A., Schaefer E.J. (1992). Plasma docosahexaenoic acid levels in various genetic forms of retinitis pigmentosa. Investig. Ophthalmol. Vis. Sci..

[91] Singh M. (2005). Essential Fatty Acids, DHA and Human Brain. Indian J. Pediatr..

[92] Minihane A.M. (2016). Impact of Genotype on EPA and DHA Status and Responsiveness to Increased Intakes. Nutrients.

[93] de la Garza Puentes A., Montes Goyanes R., Chisaguano Tonato A.M., Torres-Espinola F.J., Arias Garcia M., de Almeida L., Bonilla Aguirre M., Guerendiain M., Castellote Bargallo A.I., Segura Moreno M. (2017). Association of maternal weight with FADS and ELOVL genetic variants and fatty acid levels- The PREOBE follow-up. PLoS ONE.

[94] Gonzalez-Casanova I., Rzehak P., Stein A.D., Garcia Feregrino R., Rivera Dommarco J.A., Barraza-Villarreal A., Demmelmair H., Romieu I., Villalpando S., Martorell R. (2016). Maternal single nucleotide polymorphisms in the fatty acid desaturase 1 and 2 coding regions modify the impact of prenatal supplementation with DHA on birth weight. Am. J. Clin. Nutr..

[95] Xie L., Innis S.M. (2008). Genetic variants of the FADS1 FADS2 gene cluster are associated with altered (n-6) and (n-3) essential fatty acids in plasma and erythrocyte phospholipids in women during pregnancy and in breast milk during lactation. J. Nutr..

[96] Monthe-Dreze C., Penfield-Cyr A., Smid M.C., Sen S. (2018). Maternal Pre-Pregnancy Obesity Attenuates Response to Omega-3 Fatty Acids Supplementation During Pregnancy. Nutrients.

[97] Bernhard W., Raith M., Koch V., Maas C., Abele H., Poets C.F., Franz A.R. (2016). Developmental changes in polyunsaturated fetal plasma phospholipids and feto-maternal plasma phospholipid ratios and their association with bronchopulmonary dysplasia. Eur. J. Nutr..

[98] Clandinin M.T., Chappell J.E., Leong S., Heim T., Swyer P.R., Chance G.W. (1980). Intrauterine fatty acid accretion rates in human brain: Implications for fatty acid requirements. Early Hum. Dev..

[99] van Houwelingen A.C., Foreman-van Drongelen M.M., Nicolini U., Nicolaides K.H., Al M.D., Kester A.D., Hornstra G. (1996). Essential fatty acid status of fetal plasma phospholipids: Similar to postnatal values obtained at comparable gestational ages. Early Hum. Dev..

[100] Kuipers R.S., Luxwolda M.F., Offringa P.J., Boersma E.R., Dijck-Brouwer D.A., Muskiet F.A. (2012). Fetal intrauterine whole body linoleic, arachidonic and docosahexaenoic acid contents and accretion rates. Prostaglandins Leukot Essent Fat. Acids.

[101] Kuipers R.S., Luxwolda M.F., Sango W.S., Kwesigabo G., Dijck-Brouwer D.A., Muskiet F.A. (2011). Maternal DHA equilibrium during pregnancy and lactation is reached at an erythrocyte DHA content of 8 g/100 g fatty acids. J. Nutr..

[102] Carlson S.E., Colombo J., Gajewski B.J., Gustafson K.M., Mundy D., Yeast J., Georgieff M.K., Markley L.A., Kerling E.H., Shaddy D.J. (2013). DHA supplementation and pregnancy outcomes. Am. J. Clin. Nutr..

[103] Guesnet P., Alessandri J.M. (2011). Docosahexaenoic acid (DHA) and the developing central nervous system (CNS)—Implications for dietary recommendations. Biochimie.

[104] Rogers L.K., Valentine C.J., Keim S.A. (2013). DHA supplementation: Current implications in pregnancy and childhood. Pharm. Res..

[105] Harris W.S., Baack M.L. (2015). Beyond building better brains: Bridging the docosahexaenoic acid (DHA) gap of prematurity. J. Perinatol..

[106] Koletzko B., Lien E., Agostoni C., Bohles H., Campoy C., Cetin I., Decsi T., Dudenhausen J.W., Dupont C., Forsyth S. (2008). The roles of long-chain polyunsaturated fatty acids in pregnancy, lactation and infancy: Review of current knowledge and consensus recommendations. J. Perinat. Med..

[107] Mulder K.A., Elango R., Innis S.M. (2018). Fetal DHA inadequacy and the impact on child neurodevelopment: A follow-up of a randomised trial of maternal DHA supplementation in pregnancy. Br. J. Nutr..

[108] Rees A., Sirois S., Wearden A. (2019). Prenatal maternal docosahexaenoic acid intake and infant information processing at 4.5 mo and 9 mo: A longitudinal study. PLoS ONE.

[109] Maslova E., Rifas-Shiman S.L., Olsen S.F., Gillman M.W., Oken E. (2018). Prenatal n-3 long-chain fatty acid status and offspring metabolic health in early and mid-childhood: Results from Project Viva. Nutr. Diabetes.

[110] Ramakrishnan U., Gonzalez-Casanova I., Schnaas L., DiGirolamo A., Quezada A.D., Pallo B.C., Hao W., Neufeld L.M., Rivera J.A., Stein A.D. (2016). Prenatal supplementation with DHA improves attention at 5 y of age: A randomized controlled trial. Am. J. Clin. Nutr..

[111] Crozier S.R., Sibbons C.M., Fisk H.L., Godfrey K.M., Calder P.C., Gale C.R., Robinson S.M., Inskip H.M., Baird J., Harvey N.C. (2018). Arachidonic acid and DHA status in pregnant women is not associated with cognitive performance of their children at 4 or 6–7 years. Br. J. Nutr..

[112] Hoffman D.R., Theuer R.C., Castañeda Y.S., Wheaton D.H., Bosworth R.G., O’Connor A.R., Morale S.E., Wiedemann L.E., Birch E.E. (2004). Maturation of visual acuity is accelerated in breast-fed term infants fed baby food containing DHA-enriched egg yolk. J. Nutr..

[113] Meldrum S.J., D’Vaz N., Simmer K., Dunstan J.A., Hird K., Prescott S.L. (2012). Effects of high-dose fish oil supplementation during early infancy on neurodevelopment and language: A randomised controlled trial. Br. J. Nutr..

[114] Treen M., Uauy R.D., Jameson D.M., Thomas V.L., Hoffman D.R. (1992). Effect of docosahexaenoic acid on membrane fluidity and function in intact cultured Y-79 retinoblastoma cells. Arch. Biochem. Biophys..

[115] Hyman B.T., Spector A.A. (1982). Choline uptake in cultured human Y79 retinoblastoma cells: Effect of polyunsaturated fatty acid compositional modifications. J. Neurochem..

[116] Machova E., Novakova J., Lisa V., Dolezal V. (2006). Docosahexaenoic acid supports cell growth and expression of choline acetyltransferase and muscarinic receptors in NG108-15 cell line. J. Mol. Neurosci..

[117] Machova E., Malkova B., Lisa V., Novakova J., Dolezal V. (2006). The increase of choline acetyltransferase activity by docosahexaenoic acid in NG108-15 cells grown in serum-free medium is independent of its effect on cell growth. Neurochem. Res..

[118] da Costa K.-A., Rai K.S., Craciunescu C.N., Parikh K., Mehedint M.G., Sanders L.M., McLean-Pottinger A., Zeisel S.H. (2010). Dietary Docosahexaenoic Acid Supplementation Modulates Hippocampal Development in the Pemt−/− Mouse. J. Biol. Chem..

[119] Pynn C.J., Henderson N.G., Clark H., Koster G., Bernhard W., Postle A.D. (2011). Specificity and rate of human and mouse liver and plasma phosphatidylcholine synthesis analyzed in vivo. J. Lipid Res..

[120] van Deijk A.F., Broersen L.M., Verkuyl J.M., Smit A.B., Verheijen M.H.G. (2017). High Content Analysis of Hippocampal Neuron-Astrocyte Co-cultures Shows a Positive Effect of Fortasyn Connect on Neuronal Survival and Postsynaptic Maturation. Front. Neurosci..

[121] Fourrier C., Remus-Borel J., Greenhalgh A.D., Guichardant M., Bernoud-Hubac N., Lagarde M., Joffre C., Laye S. (2017). Docosahexaenoic acid-containing choline phospholipid modulates LPS-induced neuroinflammation in vivo and in microglia in vitro. J. Neuroinflam..

[122] Almeida P.M.D., Kamath S.U., Shenoy P.R., Bernhardt L.K., Kishore A., Rai K.S. (2018). Persistent attenuation of brain oxidative stress through aging in perinatal maternal separated rat pups supplemented with choline and docosahexaenoic acid or Clitoria ternatea aqueous root extract. Folia Neuropathol..

[123] van Wijk N., Balvers M., Cansev M., Maher T.J., Sijben J.W., Broersen L.M. (2016). Dietary Crude Lecithin Increases Systemic Availability of Dietary Docosahexaenoic Acid with Combined Intake in Rats. Lipids.

[124] Rajarethnem H.T., Megur Ramakrishna Bhat K., Jc M., Kumar Gopalkrishnan S., Mugundhu Gopalram R.B., Rai K.S. (2017). Combined Supplementation of Choline and Docosahexaenoic Acid during Pregnancy Enhances Neurodevelopment of Fetal Hippocampus. Neurol Res. Int..

[125] Zicker S.C., Jewell D.E., Yamka R.M., Milgram N.W. (2012). Evaluation of cognitive learning, memory, psychomotor, immunologic, and retinal functions in healthy puppies fed foods fortified with docosahexaenoic acid-rich fish oil from 8 to 52 weeks of age. J. Am. Vet. Med. Assoc..

[126] Lima H.K., Lin X., Jacobi S.K., Man C., Sommer J., Flowers W., Blikslager A., Gonzalez L., Odle J. (2018). Supplementation of Maternal Diets with Docosahexaenoic Acid and Methylating Vitamins Impacts Growth and Development of Fetuses from Malnourished Gilts. Curr. Dev. Nutr..

[127] Kwan S.T.C., King J.H., Yan J., Wang Z., Jiang X., Hutzler J.S., Klein H.R., Brenna J.T., Roberson M.S., Caudill M.A. (2017). Maternal Choline Supplementation Modulates Placental Nutrient Transport and Metabolism in Late Gestation of Mouse Pregnancy. J. Nutr..

[128] Maas C., Franz A.R., Shunova A., Mathes M., Bleeker C., Poets C.F., Schleicher E., Bernhard W. (2017). Choline and polyunsaturated fatty acids in preterm infants’ maternal milk. Eur. J. Nutr..

[129] Ferre C., Callaghan W., Olson C., Sharma A., Barfield W. (2016). Effects of Maternal Age and Age-Specific Preterm Birth Rates on Overall Preterm Birth Rates—United States, 2007 and 2014. Mmwr. Morb. Mortal. Wkly. Rep..

[130] Behrman R.E., Butler A.S., Institute of Medicine Committee on Understanding Premature Birth, Assuring Healthy Outcomes (2007). The National Academies Collection: Reports funded by National Institutes of Health. Preterm Birth: Causes, Consequences, and Prevention.

[131] Bernhard W., Böckmann K., Maas C., Mathes M., Hovelmann J., Shunova A., Hund V., Schleicher E., Poets C.F., Franz A.R. (2019). Combined choline and DHA supplementation: A randomized controlled trial. Eur. J. Nutr..

[132] West A.A., Yan J., Jiang X., Perry C.A., Innis S.M., Caudill M.A. (2013). Choline intake influences phosphatidylcholine DHA enrichment in nonpregnant women but not in pregnant women in the third trimester. Am. J. Clin. Nutr..

[133] West A.A., Shih Y., Wang W., Oda K., Jaceldo-Siegl K., Sabate J., Haddad E., Rajaram S., Caudill M.A., Burns-Whitmore B. (2014). Egg n-3 fatty acid composition modulates biomarkers of choline metabolism in free-living lacto-ovo-vegetarian women of reproductive age. J. Acad. Nutr. Diet..

[134] da Costa K.A., Sanders L.M., Fischer L.M., Zeisel S.H. (2011). Docosahexaenoic acid in plasma phosphatidylcholine may be a potential marker for in vivo phosphatidylethanolamine N-methyltransferase activity in humans. Am. J. Clin. Nutr..

[135] McNamara R.K., Jandacek R., Tso P., Weber W., Chu W.J., Strakowski S.M., Adler C.M., Delbello M.P. (2013). Low docosahexaenoic acid status is associated with reduced indices in cortical integrity in the anterior cingulate of healthy male children: A 1H MRS Study. Nutr. Neurosci..

[136] Cheatham C.L., Sheppard K.W. (2015). Synergistic Effects of Human Milk Nutrients in the Support of Infant Recognition Memory: An Observational Study. Nutrients.

[137] Jackson K.H., Harris W.S. (2018). A Prenatal DHA Test to Help Identify Women at Increased Risk for Early Preterm Birth: A Proposal. Nutrients.

